# Clinically Actionable Insights into Initial and Matched Recurrent Glioblastomas to Inform Novel Treatment Approaches

**DOI:** 10.1155/2019/4878547

**Published:** 2019-12-31

**Authors:** H. P. Ellis, C. E. McInerney, D. Schrimpf, F. Sahm, A. Stupnikov, M. Wadsley, C. Wragg, P. White, K. M. Prise, D. G. McArt, K. M. Kurian

**Affiliations:** ^1^Brain Tumour Research Centre, University of Bristol, Bristol, UK; ^2^Centre for Cancer Research and Cell Biology, Queen's University, Belfast, UK; ^3^Department of Neuropathology, Institute of Pathology, Ruprecht-Karls-University Heidelberg, Heidelberg, Germany; ^4^Department of Oncology, School of Medicine, Johns Hopkins University, Baltimore, MD 21287, USA; ^5^Bristol Genetics Laboratory, North Bristol NHS Trust, Bristol, UK; ^6^Applied Statistics Group, University of the West of England, Bristol, UK

## Abstract

Glioblastoma is the most common primary adult brain tumour, and despite optimal treatment, the median survival is 12–15 months. Patients with matched recurrent glioblastomas were investigated to try to find actionable mutations. Tumours were profiled using a validated DNA-based gene panel. Copy number variations (CNVs) and single nucleotide variants (SNVs) were examined, and potentially pathogenic variants and clinically actionable mutations were identified. The results revealed that glioblastomas were *IDH*-wildtype (*IDH*^WT^; *n* = 38) and *IDH*-mutant (*IDH*^MUT^; *n* = 3). SNVs in *TSC2*, *MSH6*, *TP53*, *CREBBP*, and *IDH1* were variants of unknown significance (VUS) that were predicted to be pathogenic in both subtypes. *IDH*^WT^ tumours had SNVs that impacted RTK/Ras/PI(3)K, p53, WNT, SHH, NOTCH, Rb, and G-protein pathways. Many tumours had *BRCA1/2* (18%) variants, including confirmed somatic mutations in haemangioblastoma. *IDH*^WT^ recurrent tumours had fewer pathways impacted (RTK/Ras/PI(3)K, p53, WNT, and G-protein) and CNV gains (*BRCA2*, *GNAS*, and *EGFR*) and losses (*TERT* and *SMARCA4*). *IDH*^MUT^ tumours had SNVs that impacted RTK/Ras/PI(3)K, p53, and WNT pathways. VUS in *KLK1* was possibly pathogenic in *IDH*^MUT^. Recurrent tumours also had fewer pathways (p53, WNT, and G-protein) impacted by genetic alterations. Public datasets (TCGA and GDC) confirmed the clinical significance of findings in both subtypes. Overall in this cohort, potentially actionable variation was most often identified in *EGFR*, *PTEN*, *BRCA1/2*, and *ATM*. This study underlines the need for detailed molecular profiling to identify individual GBM patients who may be eligible for novel treatment approaches. This information is also crucial for patient recruitment to clinical trials.

## 1. Introduction

Gliomas are the largest group of intrinsic brain tumours with age adjusted incidence rates ranging from 4.67 to 5.73 per 100,000, causing more years of life lost compared with other cancers [1, 2]. Glioblastoma (GBM) is the most malignant glioma and is classified molecularly as *IDH*-wildtype and *IDH*-mutant GBM [3–10]. During gliomagenesis, an array of genetic alterations may cause the dysregulation of cell growth signalling and cell cycle pathways [6, 11–15]. In particular, mutations in *RTKs* (receptor tyrosine kinases) and/or loss of *PTEN* (phosphatase and tensin homolog) alter the *PI3K* (phospinositide 3-kinase)/*AKT* cell growth pathway [11]. Further mutations in *CDKN2A* or *CDK4* (cyclin-dependent kinase) lead to uncontrolled progression of the cell cycle, as do mutations in *TP53* [16]. Neural stem cells in the subventricular zone may harbour recurrent driver somatic mutations that are shared with the tumour bulk (e.g., *P53*, *PTEN*, *EGFR*, and *TERT*) [17]. Telomerase (reactivation or reexpression) can occur in *IDH* wildtype and mutant GBMs driven either by telomerase reverse transcriptase (*TERT*) promoter mutations or other mechanisms [8, 18]. The current standard-of-care for glioblastomas remains as maximal safe surgical resection with concurrent radiotherapy and temozolomide (TMZ) chemotherapy (Stupp protocol) [19, 20]. Personalised therapies remain promising although trials have been unsuccessful to date [21–23]. For example, dysregulated *PI3K* and *RTKs* (*EGFR*, *MET*, *PDGFR*, *FGFR*, and *BRAF*) genes have been targeted with various small molecules, antibodies, and inhibitors [24–29]. To date, entry to clinical trials for GBM has not been based on a detailed molecular analysis of an individual patient's tumour using high throughput sequencing (HTS). HTS-based molecular diagnostics can aid the detection of genetic alterations, information required for personalised medicine [30, 31]. Herein, initial and matched recurrent glioblastomas were examined using HTS with a validated DNA-based diagnostic panel. Potentially pathogenic variants and clinically actionable mutations were identified in different GBM subtypes. Findings were validated using TCGA-GBM and GDC datasets.

## 2. Materials and Methods

### 2.1. Clinical Specimens

Ethical approval was given by Brain Tumour Bank South West and Brain UK (Ref: 14/010). All patients had been treated using the Stupp protocol [19]. A total of 72 formalin-fixed paraffin-embedded (FFPE) samples from 54 patients were identified (2009–2014). Only FFPE slides with >30% tumour cells available for macrodissection were selected. Samples lacking cellularity or excessively necrotic were excluded. Following quality control, 67 samples for 46 patients and 19 with matched recurrent samples available were identified. Of these, a total of 49 samples were successfully sequenced for 41 patients (21 males; 20 females; mean age 55 years, range 16–78 years; see Tables 1 and [Supplementary-material supplementary-material-1]). Matched initial and recurrent tissue samples were analysed for 8 patients (2 males; 6 females). Recurrent tumours all occurred locally to the initial tumour. Anonymised patient cases in the GBM cohort were numbered 1–11, 16–41, and 43–46, and “*a*” and “*b*” indicated initial and recurrent tumour samples, respectively ([Supplementary-material supplementary-material-1]).

### 2.2. HTS Neuro-Oncology Gene Panel

A published HTS DNA-based panel that uses targeted enrichment to examine exonic, selected intronic and promoter regions of 130 clinically relevant neuro-oncology genes was utilised (see [Supplementary-material supplementary-material-1]) [30]. The diagnostic panel has been optimised for use either with fresh-frozen or FFPE tissue. Validation studies of the HTS panel analysing ∼200 single nucleotide variants (SNVs), gene fusions, and copy number variants (CNVs) showed 98% concordance with single marker tests [30]. Using the HTS panel, genetic alterations in tumours were characterized, and *TERT* promoter and *IDH1*/*2* status confirmed.

### 2.3. DNA Extraction, HTS Library Preparation, Sequencing, and Analysis

Slides were deparaffinised and rehydrated using xylene and ethanol and left to dry. Tissue sections were then microdissected and placed into 180 uL ATL buffer. DNA was extracted from tissue sections (10 × 10 *μ*m) according to manufacturer's instructions using the QIAamp DNA FFPE Tissue Kit (Qiagen, Manchester, UK). Following assessment of DNA quality and quantity, libraries were prepared using 200 ng of genomic DNA with an optical density 260/280 ratio between 1.8 and 2.0. Libraries were constructed using the SureSelect^XT^ Target Enrichment System for Illumina Paired-End Multiplexed Sequencing Library protocol (Agilent). PCR master mixes were prepared using the SureSelect^XT^ Library Prep Kit ILM following manufacturer's guidelines. In accordance with Illumina guidelines, libraries with a concentration of 4 nM were diluted to 20 pM, denatured, and sequenced on a NextSeq 500 (Illumina). HTS data were analysed following the pipeline described by Sahm et al. [30]. In brief, raw reads were demultiplexed, converted to *fastq*, quality checked, and manually trimmed when necessary. Paired-end reads were aligned to the human genome (version GRch37; hg19), and duplicate sequences were removed.

### 2.4. CNV Analysis in the GBM Cohort

CNVs were investigated using a coverage analysis. The ratio of on- and off-target reads, coverage per target region, and mean coverage per sample were estimated using the *R* package *TEQC* [32]. Measures provided an estimate of read depth, as the number of reconstructed strands across a region of interest, and this was utilised for CNV estimation of genes. Data normalisation and CNV comparison to a reference control were made using the *R* package *seqCNA* [33]. This method has previously been validated with 100% concordance for 47 GBM cases using 450 k data [30]. Potential CNV gain or loss is indicated by deviations from a proportional read depth of 50%, considered a normal gene copy number.

### 2.5. SNV Analysis in the GBM Cohort

Variant calling followed a modified pipeline, as described by Sahm et al. [30]. In brief, variants were called using *SAMtools mpileup* [34]. Variant calls were then filtered by (a) read depth ≥ 40, (b) genotype quality ≥ 99, (c) minimum allele frequency set at 10, and (d) at least 10% read coverage from each strand using the *R* package *VariantAnnotation* [35]. *TERT* promoter position calls were not filtered due to their low detection rate because of difficulties with their amplification as a GC-rich region [30]. Nonsynonymous filtered variants were annotated with the most up to date information including dbSNP and COSMIC identifiers using the online tool *wANNOVAR* [36]. Matched normal tissue was unavailable for comparison for the identification of germline mutations. Thus, to try to discern pathogenic from benign variants, the frequency of a variant in the general population was used as a key criterion in their clinical interpretation to try to exclude germline mutations. SNVs were filtered to those with a frequency of ≥0.01 in the 1,000 Genomes database and ≥0.05 in the Genome Aggregation Database (gnomAD), previously known as the Exome Aggregation Consortium database. gnomAD warehouses whole genome sequences from 15,496 unrelated individuals [37]. As the ethnicity of patients in the GBM cohort was unknown, SNV frequencies were compared to overall frequencies (rather than regional) of both databases. Filtered SNVs impacting genes were categorised into biological pathways using *GeneCards* [38]. SNVs occurring in the potentially clinically actionable genes: *EGFR*, *PTEN*, *CDKN2A*, *RB1*, *TP53*, *ATM*, *ATR*, *MSH6*, *PDGFRA*, *PIK3CA*, *PIK3R1*, *SMO*, *PTCH1*, *BRCA1*, *BRCA2,* and *BRAF*, were quantified in the initial and matched recurrent tumours. Further filtering was applied to SNV results to try to identify variants of unknown significance (VUS) that are possibly pathogenic and underpin gliomagenesis. VUS considered to be possibly pathogenic, were those that had no frequency recorded in the 1,000 Genomes database, and were predicted to be damaging by both *LJB SIFT* and *FATHMM-MKL* software [39]. All genomic positions listed for SNVs identified by this study are from the human genome version GRch37.

### 2.6. VUS and CNV Analysis in the TCGA-GBM and GDC Datasets

VUS identified as possibly pathogenic mutations in the GBM cohort were further investigated for supporting evidence of their clinical significance using TCGA-GBM and GDC datasets. Frequencies of cases with mutations in genes were investigated in the GDC data portal. Abundance of mutations and copy number alterations within the TCGA-GBM dataset was visualised as an oncoprint plot generated using *GlioVis*, a data visualisation tool for brain tumour datasets [40].

### 2.7. Survival Analyses of *IDH*-Wildtype Glioblastomas

A Cox proportional hazard regression analysis was implemented to determine the relationship between the total number of SNVs (median split) and overall survival. *MGMT* methylated and unmethylated GBMs were investigated separately. Survival analyses and plotting of results as Kaplan–Meier graphs were carried out using *R* software [41]. Of the 41 patients, univariate survival analysis was carried out on the 33 *IDH*-wildtype patients only. Omitted patients included the three *IDH*^MUT^ patients and a further five patients lacking survival information.

## 3. Results

### 3.1. Overview of Genomic Profiling of Glioblastoma Tumours and *IDH* Status

In all, 49 samples from 41 patients including 8 matched samples were genomically profiled (Tables 1 and [Supplementary-material supplementary-material-1]). Results could not be obtained for 5 initial and 13 recurrent samples from 11 patients, giving a sequencing failure rate of ∼22%. SNVs were not identified in 5 samples (9%). Recurrent tumour samples were necrotic with low cellularity, which probably impacted DNA quality and sequencing success. Majority of tumours were *IDH*-wildtype (38/41; 93%) with the exception of three cases (*8*, *35*, and *39*) that were *IDH*-mutant ([Supplementary-material supplementary-material-1]). Cases 8, 35, and 39 had a C to T mutation located at the *IDH1* diagnostic hotspot R132 (Chr2: 209113112; GRCh37). Only one other case (6a) had an *IDH1* mutation located at Chr2: 209108284 (GRCh37). This mutation was 4,828 bp upstream of the diagnostic hotspot (R132); hence, case 6a was considered *IDH*-wildtype. One case had an *IDH2* mutation (Chr15: 90627553); however, this did not coincide with known somatic mutations located at 15q26.1 codons R140 (Chr15: 90631934) and R172 (Chr15: 90631837). *TERT* mutations were observed in *IDH* wildtype initial (Chr5: 1254594; Chr5: 1294166) and recurrent tumours (Chr5: 1,254,594); however, none coincided with known somatic mutations in promoter regions at the C228 (Chr5: 1,295,228) and C250 loci (Chr5: 1,295,250; hg19).

### 3.2. SNVs Detected in Initial and Recurrent *IDH*^WT^ Glioblastomas

**Figure 1:**
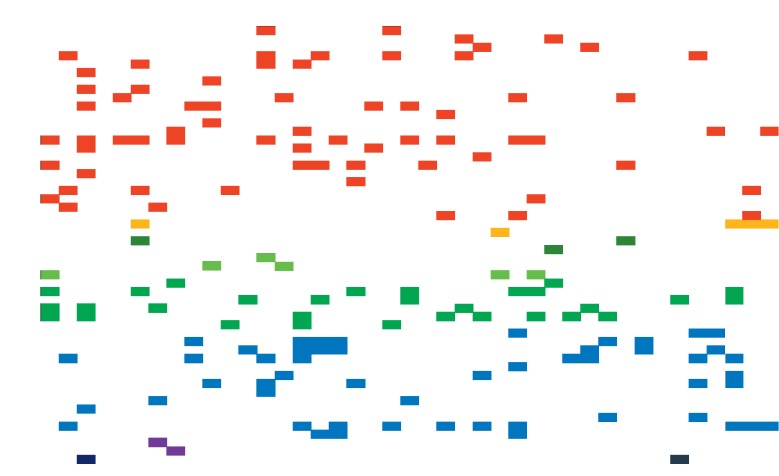
Summary of the genes identified with SNVs in *IDH*^*WT*^ (*n* = 38) and *IDH*^*MUT*^ diffuse tumours (*n* = 3; cases 8a, 35a, and 39a). Genes are arranged hierarchically within their pathways for the RTK/Ras/PI(3)K (red), IDH (yellow), NOTCH, SHH, and WNT signalling (variations of green), p53 (blue), Rb (purple), and G-proteins (dark blue) pathways. Numbers across the top axis denote the patient identifier.

**Figure 2:**
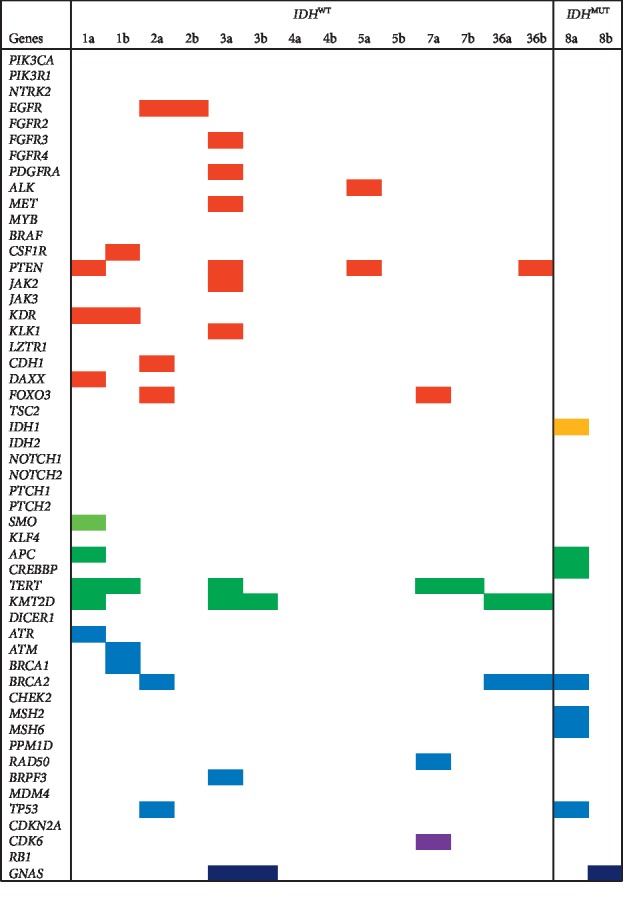
Summary of the genes identified with SNVs in matched initial and recurrent *IDH*^*WT*^ (*n* = 7) and *IDH*^*MUT*^ diffuse tumours (*n* = 1; case 8). Genes are arranged hierarchically within their pathways for the RTK/Ras/PI(3)K (red), IDH (yellow), NOTCH, SHH, and WNT signalling (variations of green), p53 (blue), Rb (purple), and G-proteins (dark blue) pathways. Numbers across the top axis denote the patient identifier; “a” and “b” indicate initial and recurrent tumours, respectively.

A total of 134 nonsynonymous and three stop-gain SNVs were detected from initial (*n* = 125; [Supplementary-material supplementary-material-1]) and recurrent *IDH*^WT^ tumours (*n* = 12; [Supplementary-material supplementary-material-1]). Including *IDH*1/2 mutations, SNVs affected 52 genes across nine biological pathways during the different phases of gliomagenesis (Figures 1 and 2; Tables 2 and 3). Majority of initial tumours had SNVs in a gene in the RTK/Ras/PI(3)K pathways (79%; 30/38) followed by the p53 DNA damage repair pathway (61%; 23/38). Two stop-gain SNVs were identified from the p53 genes *MSH2* (Chr2: 47705428; rs63751155) and *TP*53 (Chr17: 7579315; COSM326717; COSM3388232; COSM326718; COSM3388233; COSM326716) in initial tumours; both variants were predicted to be pathogenic by *FATHMM-MKL* ([Supplementary-material supplementary-material-1]). A large proportion of initial *IDH*^WT^ tumours had SNVs in the p53 pathway genes *BRCA*1 (18%; 7/38) and *BRCA*2 (18%; 7/38; Table 4). Six *BRCA*1 variants were detected including a confirmed somatic mutation in adenocarcinoma (COSM6612515; Chr17: 41244952) [42]. Six *BRCA*2 variants were detected including confirmed somatic mutations in haemangioblastoma (COSM3753648, Chr13: 32914236; COSM5019704, Chr13: 32953549) [43]. Over half of initial *IDH*^WT^ tumours had an SNV in a WNT signalling pathway gene (58%; 22/38). Multiple variants (*n*) were detected for the WNT genes *KMT2D/MLL2* (*7*), *CREBBP* (*4*), *DICER1* (*3*), *APC* (*3*), *TERT* (*2*), and *KLF4* (*2*). *IDH*^WT^ tumours also showed variation in SHH (16%; 6/38) and NOTCH (8%; 3/38) pathways. A small proportion of initial tumours had SNVs in the G-protein gene, *GNAS* (5%; 2/38), *IDH1/2* (5%; 2/38), and the Rb-specific cell-cycle regulation genes *CDK6* and *RB1* (5%; 2/38). The *RB1* variant was a stop-gain SNV (Chr13: 48953735), but it was not pathogenic. Among *IDH*^WT^ tumours, 40 SNVs in 21 genes were VUS that were predicted to be functionally damaging (Tables 3 and [Supplementary-material supplementary-material-1]). Potentially pathogenic VUS impacted *IDH1* and genes in the p53 (*ATM*, *BRCA1*, *CHEK2*, *MSH6*, *PPM1D*, and *TP53*), RTK/Ras/PI(3)K (*BRAF*, *DAXX*, *EGFR*, *FGFR2*, *JAK2*, *MYB*, *PIK3CA*, *PIK3R1*, *TSC2*, and *PTEN*), SHH (*PTCH1* and *SMO*), and WNT pathways (CREBBP). Two-thirds of initial *IDH*^WT^ tumours (63%; 24/38) harboured potentially actionable variation most frequently in *PTEN* (29%; 11/38), followed by *BRCA1* (18%; 7/38), *BRCA2* (18%; 7/38), *TP53* (18%; 7/38), *EGFR* (16%; 6/38), *ATM* (16%; 6/38), and *ATR* (8%; 3/38; see Table 4). Recurrent *IDH*^WT^ tumours had SNVs in genes in the RTK/Ras/PI(3)K (43%; 3/7), WNT signalling (57%; 4/7), and p53 pathways (29%) in the genes *BRCA1* (14%; 1/7) and *BRCA2* (14%; 1/7) and *GNAS* (14%; 1/7). *IDH*^WT^ recurrent tumours were not mutated in NOTCH, SHH, Rb, or *IDH* genes (Figure 2 and [Supplementary-material supplementary-material-1]). In the matched initial tumour, 16 genes showed variation, four of which were also mutated in the recurrent tumour. An additional three SNVs were recorded only in the recurrent tumour in *CSF1R*, *ATM,* and *BRCA1*. Possibly pathogenic VUS were identified in *PTEN* in recurrent *IDH*^WT^ tumours. Almost half of recurrent *IDH*^WT^ tumours (43%; 3/7) harboured at least one potentially actionable variation in the genes *EGFR* (14%; 1/7), *PTEN* (14%; 1/7), *BRCA1* (14%; 1/7), *BRCA2* (14%; 1/7), and *ATM* (14%; 1/7; Figure 2 and Table 4).

### 3.3. SNVs Detected in Initial and Recurrent *IDH*^MUT^ Glioblastomas

SNVs detected in *IDH*^MUT^ initial (*n* = 12) and recurrent tumours (*n* = 1; Tables [Supplementary-material supplementary-material-1], and [Supplementary-material supplementary-material-1]) impacted *IDH*1 and 10 genes across 5 biological pathways (Figures 1 and 2; Table 2). Majority of initial tumours had SNVs in genes in the RTK/Ras/PI(3)K (66%; 2/3), followed by p53 (100%; 3/3) and WNT signalling pathway (33%; 1/3). All initial *IDH*^MUT^ tumours (100%; 3/3) harboured at least one potentially actionable variation in *TP53* (100%; 3/3), *BRCA2* (33%; 1/3), and *MSH6* (33%; 1/3; Table 4). Just 7 SNVs in 6 genes were VUS that were possibly pathogenic in *IDH*^MUT^ initial tumours. These included *IDH1* and the p53 pathway genes *MSH6* and *TP53* and the RTK/Ras/PI(3)K genes *KLK1* and *TSC2* and the *CREBBP* gene in the WNT pathway (Table 3). The *KLK1* variant was potentially pathogenic in *IDH*^MUT^ but not in *IDH*^WT^. The recurrent *IDH*^MUT^ tumour had SNVs in p53, WNT signalling, and G-protein pathway genes. Matched analysis revealed that seven genes had SNVs in the initial that were not observed in the recurrent tumour (Figure 2). The recurrent tumour had SNVs in one gene not recorded in the initial (*GNAS*). No genes had SNVs that were potentially actionable in the recurrent *IDH*^MUT^ tumour (Table 4).

### 3.4. CNVs in *IDH*^WT^ and *IDH*^MUT^ Glioblastomas

CNVs were detected in *IDH*^WT^ tumours only ([Supplementary-material supplementary-material-1]). The results for CNVs in the corresponding genes in TCGA-GBM are presented in [Supplementary-material supplementary-material-1]. For sample 36, there appears to be a hemizygous deletion in *BRCA*2 in the initial, but a CNV gain in the recurrent tumour. Both trends were identified in TCGA-GBM, but predominantly *BRCA2* had shallow deletions. There were CNV gains in *GNAS* for recurrent sample *3b*. TCGA-GBM results also predominantly indicate CNV gains for *GNAS*. In recurrent samples *1b* and *7b*, *TERT* appeared to have hemizygous deletions. TCGA-GBM had both *TERT* CNV losses and gains with no predominant trend evident. For *SMARCA4*, there appears to be a CNV gain in initial sample *1* but a hemizygous deletion in the recurrent sample. TCGA-GBM had mostly CNV gains with some losses for *SMARCA4*. Significant CNV gains in *EGFR* were observed for initial and recurrent sample *2* and similarly in TCGA-GBM cases.

### 3.5. Investigation of the Corresponding Genes (with Mutations and CNVs in the GBM Cohort) in the TCGA-GBM and GDC Datasets

The results of investigations in the TCGA-GBM and GDC datasets for the 21 genes identified with VUS that were possibly pathogenic in the GBM cohort are presented in [Supplementary-material supplementary-material-1]. A summary of SNVs identified from those corresponding genes in the TCGA-GBM dataset is provided in [Supplementary-material supplementary-material-1]. TCGA-GBM cases in the mutation data included 6 verified and 2 ambiguous *IDH*-mutant individuals; however, majority of cases are unannotated. *PTEN* was the gene most impacted by mutations (34.86%) and shallow or deep deletions ([Supplementary-material supplementary-material-1]; [Supplementary-material supplementary-material-1]). *EGFR* had mutations (26.97%) and CNV gains. *FGFR2* (1.53%), *JAK2* (1.27%), *MYB* (1.27%), and *ATM* (2.04%) had fewer mutations and mostly shallow or deep deletions. Both *BRAF* (2.54%) and *SMO* (1.02%) had fewer mutations and mostly low level CNV gains. *TP53* (31.55%), *PIK3CA* (10.18%), and *PIK3R1* (10.94%) had relatively high mutations and a mixture of CNV gains and deletions. *IDH1* (6.62%), *BRCA1* (2.8%), *PTCH1* (3.56%), *CREBBP* (3.56%), *MSH6* (3.05%), *DAXX* (2.29%), *TSC2* (2.04%), *PPM1D* (1.78%), *KLK1* (0.51%), and *CHEK2* (0.25%) had low rate of mutations and a mixture of CNV low level gains and losses. *BRCA1* (2.8%) had low rate of mutations and both CNV low level gains and shallow or deep deletions. The results for the 12 NOTCH, SHH, and WNT pathway genes identified to be impacted in the GBM cohort investigated in the TCGA-GBM and GDC datasets are presented in [Supplementary-material supplementary-material-1] and [Supplementary-material supplementary-material-1]. The WNT pathway genes *DICER1* (2.29%), *KLF4* (0.25%), and *CREBBP* (3.56%) had mutations and CNV shallow deletions, as well as low level gains and high level amplifications. *TERT* (2.80%) and *KMT2D* (3.05%) had mutations and CNV shallow gains and losses as well as deep deletions. *APC* (4.58%) and *TCF4* (0.76%) had mutations, low level gains, and shallow deletions. The SHH genes, *PTCH1* (3.56%), *PTCH2* (1.78%), and *SMO* (1.02%) were impacted by mutations. Whilst the SMO gene had CNV gains, by comparison, the *PTCH1* and *PTCH2* genes had both CNV gains and losses. NOTCH genes, *NOTCH2* (4.07%) and *NOTCH1* (0.25%), had mutations and were impacted also by gains and losses in CNV.

### 3.6. Impact of SNV Burden on Survival in *IDH*^WT^ GBM Patients

**Figure 3:**
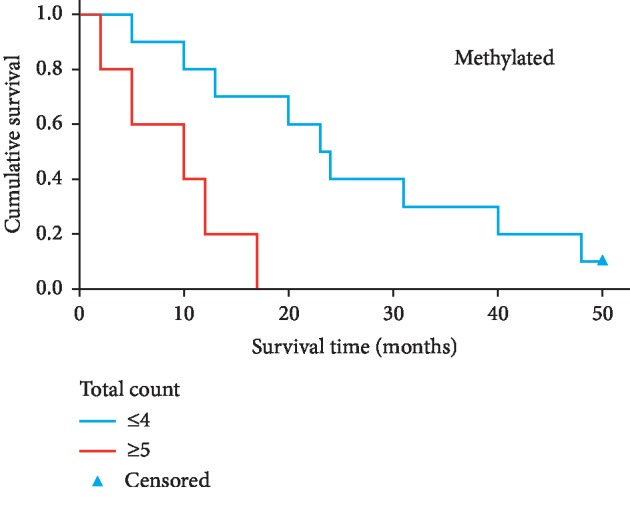
Comparison of survival for *IDH*^WT^ glioblastoma *MGMT* methylated patients with high versus low total number of tumour SNVs, based on a median split. Kaplan–Meier analysis indicates that *IDH*^*WT*^ GBM patients with a greater tumour SNV burden have significantly a shorter overall survival (*P*=0.006).

**Figure 4:**
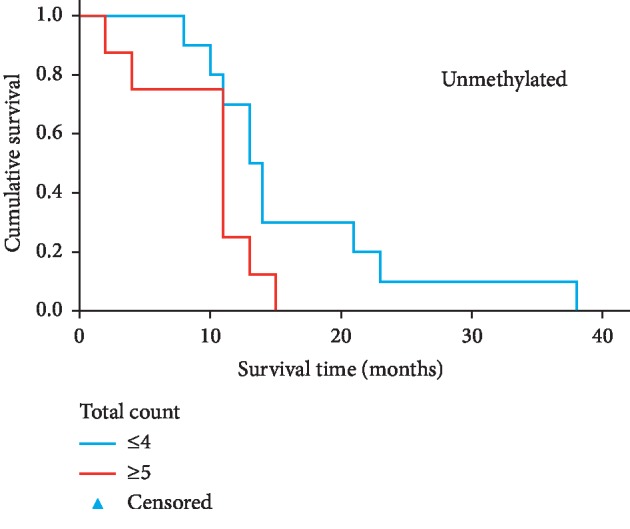
Comparison of survival for *IDH*^WT^ glioblastoma *MGMT* unmethylated patients with high versus low total number of tumour SNVs, based on a median split. Kaplan–Meier analysis indicates that *IDH*^*WT*^ GBM patients with a greater tumour SNV burden have a shorter overall survival; however, this trend was not significant (*P*=0.065).

The number of tumour SNVs was prognostic for survival in methylated GBM patients (log rank = 7.63, 95% CI = 6.90–27.10; *P* value = 0.006, two-sided). Median survival for methylated GBM with ≤ 4 SNVs was 23 months compared to a median survival of 10 months for a tumour with ≥ 5 SNVs (Figure 3; [Supplementary-material supplementary-material-1]). For unmethylated GBM patients, the number of tumour SNVs was not prognostic for survival (log rank = 3.393, 95% CI = 9.441–12.559; *P* value = 0.065). Median survival was 13 months for unmethylated GBMs with ≤ 4 SNVs, compared to a median survival of 11 months for ≥ 5 SNVs (Figure 4; [Supplementary-material supplementary-material-1]). Sample sizes were relatively small in these survival analyses; therefore, the observed trends would need to be confirmed using a larger cohort.

## 4. Discussion

The mutational landscape of the GBM subtypes in this cohort raises the possibility of new combinations of therapeutic approaches for individual GBM patients. Potentially actionable variation was most often identified in *EGFR*, *PTEN*, *BRCA1/2*, and *ATM*. These genetic alterations could be targeted by novel approaches with EGFR-targeting antibodies, tyrosine kinase inhibitors, and DNA damage repair inhibitors either singly or in combination. In particular, the *BRCA1/2* mutations raise the possibility that DNA damage repair agents may be an option for small numbers of GBM patients in combination with other agents. Administering olaparib *PARP* (poly (ADP-ribose) polymerase) inhibitor, developed for *BRCA1/2* mutated ovarian cancer, in combination with TMZ has shown promising results for treating relapsed glioblastoma patients in a phase I clinical trial (NCT01390571) [44]. However, patient selection to date has not been based on detailed molecular profiling with HTS. In this study's GBM cohort, both *IDH*^WT^ and *IDH*^MUT^ GBM had VUS that were predicted to be pathogenic in *MSH6* [45–47], *CREBBP* [48–52], *TP53* [17, 47], and *TSC2* [36–43, 53]. In particular, *MSH6* (MutS homolog 6) is a DNA mismatch-repair protein that has been identified as a putative driver gene in glioma [45, 47]. Similarly, *MSH6* may be involved in acquired resistance to alkylating agents [46]. Moreover, *CREBBP* (CREB binding protein gene*/CBP*) activates the DNA damage response and repair pathway by acetylating factors involved in base excision repair, nucleotide excision repair, nonhomologous end joining, and double-strand break repair (e.g., *PARP-1*, *H2AX*, and *NBS1*) [49].

### 4.1. *IDH*^WT^ Glioblastomas

In *IDH*^WT^ glioblastomas, SNVs impacted genes in the RTK/Ras/PI(3)K (79%), p53 (61%), WNT (58%), SHH (16%), NOTCH (8%), Rb (5%) and G-protein (5%) pathways. Potentially actionable mutations detected from initial *IDH*^WT^ tumours included *EGFR*, *PTEN*, *BRCA1*, *BRCA2*, *ATM*, and *ATR* [54–56]. Therapies for this subtype might include the *EGFR*-targeting antibodies, *EGFR*-targeting vaccines, TK inhibitors, erlotinib, and DNA damage repair inhibitors including olaparib and *ATR* inhibitors. Anti-*EGFR*-targeting antibodies to date have not shown clinical efficacy in GBM although trials are ongoing [57]. Similarly, trials of DNA damage repair inhibitors are underway, and the results are anticipated; however, patients have not been selected for these trials using molecular profiling with HTS.

Interestingly, in this cohort, a high proportion of *IDH*^WT^ tumours was impacted by *BRCA1* (18%) and *BRCA2* (18%) mutations. This trend was not observed in the TCGA-GBM dataset (2.8%; 2.3%); however, the *IDH* status of patients is not confirmed in most cases [58]. Only one variant from the GBM cohort (*BRCA1* : Ch17: 41246062) was identifiable amongst the TCGA-GBM dataset *BRCA1* (*n* = 16) and *BRCA2* (*n* = 39) variants. The well-known breast cancer specific germline mutations in *BRCA1* (185delAG; Chr17: 43124030–43124031 and 5382insC; Chr17: 43057065) and *BRCA2* (6174delT; Chr13: 32340301) were not amongst the variants identified in either the GBM cohort or the TCGA-GBM cohort. In this GBM cohort, amongst the *BRCA2* variants were confirmed somatic mutations in haemangioblastoma (*BRCA2* : COSM3753648, COSM5019704) [43], which is a rare, benign tumour that typically occurs in the cerebellum [3]. Many *IDH*^WT^ tumours had alterations impacting WNT [59–63] signalling pathway genes (58%) including *CREBBP*(4), *KLF4*(2) [64, 65], *TERT*(2) [17], and *APC*(3) [66–70]; however, targeting this pathway is currently challenging. Initial *IDH*^WT^ tumours also showed predicted pathogenic variation in NOTCH (11%) [71] and SHH (13%) pathways [72] including *PTCH1* (*PATCHED-1*) and *SMO* (*Smoothened*) [73–75]. The Hedgehog antagonist GDC-0449 (vismodegib) has been trialled in recurrent GBM (NCT00980343) and childhood brain tumours with varying success to date.

### 4.2. Recurrent *IDH*^WT^ Glioblastomas

Interestingly in this cohort, no tumours exhibited a TMZ-induced hypermutated phenotype. Tumours did not have mutations in TERT promoter regions. Kim et al. found that a TMZ-induced hypermutated phenotype was rare in *IDH*-wildtype primary glioblastomas [76]. Acquired resistance in glioma has been attributed to dysregulated pathways (signalling and DNA repair), persistence of cancer stem cell subpopulations, and autophagy mechanisms [77]. In this cohort, only the RTK/Ras/PI(3)K, p53 DNA damage repair, WNT signalling, and G-protein pathways were impacted by genetic alterations and not the SHH, NOTCH, and Rb pathways, despite their association with glioma resistance. Whilst fewer pathways were impacted, intertumour heterogeneity between initial and recurrent *IDH* wildtype tumours was nevertheless observed, similar to previous studies [76, 78]. Indeed, recurrent tumours can diverge to such an extent that they are no longer recognised as lineal descendants of the dominant clone identified initial at diagnosis [78, 79]. Potential signatures of *IDH*^WT^ recurrent tumour resistance included VUS that were possibly pathogenic in *PTEN*. *PTEN* mutations cause activation of the PI3K/AKT survival pathway and chemoresistance in GBM [80]. Other possible signatures of recurrent tumour resistance in this GBM cohort included CNV gains in the genes (chromosome), *BRCA2* (Chr13), *GNAS* (Guanine nucleotide-binding protein G(s) subunit alpha; Chr20), and *EGFR* (Chr7). Copy number gains are thought to impact driver genes to initiate tumourigenesis. The oncogene *EGFR* is located on chromosome 7, which frequently has CNV gains in *IDH*-wildtype glioblastomas (∼70%) [5, 6]. Gains in the chromosome 20 arm containing *GNAS* are frequently observed in pituitary brain tumours (adenomas) and may exert a mitogenic influence on the WNT signalling pathway via cAMP activation, which may provide a proliferative advantage for resistance [81]. However, *GNAS* has not been identified as a prognostic in dicator implicated in GBM [82]. CNV losses observed in the GBM cohort included *SMARCA4* (Chr19) [47] and *TERT* (Chr5). CNV losses may be concordant with gene expression downregulation [83].

### 4.3. *IDH*^MUT^ Glioblastomas

Results for *IDH*^MUT^ glioblastomas comprised three initial and one recurrent case only. Pathways impacted by genetic alterations included the RTK/Ras/PI(3)K (66%), p53 (100%), and WNT pathways (33%). Possibly pathogenic VUS identified herein included those co-mutated in both subtypes as well as *KLK1* (kallikrein1). The kallikreins *KLK6*, *KLK7,* and *KLK9* have been shown to have higher protein levels in Grade IV glioma compared to Grade III tumours and consequently may have utility as prognostic markers for patient survival [84]. All initial *IDH*^MUT^ tumour samples harboured potentially actionable variation in at least one of the genes *TP53*, *BRCA2,* and *MSH6*. The recurrent tumour had fewer pathways (p53, WNT, and G-protein) impacted by genetic alterations. Matched analysis revealed intertumour heterogeneity. The recurrent *IDH*^MUT^ tumour lacked potentially actionable variation that could be targeted. Given the small sample size for this subtype all trends reported here would need to be confirmed in a larger cohort.

## 5. Conclusion

Our study reveals that matched initial and recurrent GBM samples harbour potentially actionable variations, and these were most often identified in *EGFR*, *PTEN*, *BRCA1/2*, and *ATM*. These genetic alterations could potentially be targeted by novel approaches with EGFR-targeting antibodies, tyrosine kinase inhibitors, and DNA damage repair inhibitors either singly or in combination. This study underlines the need for detailed genetic analysis of GBM patients to identify individuals that might benefit from novel therapeutic approaches that are becoming available in the near future. This information is also important for patient recruitment to clinical trials.

## Figures and Tables

**Table 1:** Summary of the clinical data for patients genomically profiled in this study (*n* = 41). Patients with *IDH-*wildtype and *IDH*-mutant glioblastoma tumours were identified from the BRASH clinical database between 2009 and 2014.

Characteristic	*IDH*-wildtype	*IDH*-mutant
Number of patients	38	3
Age		
Mean	54	42
Median (range)	52 (16–78)	50 (19–58)
Gender		
Male	19 (50%)	2 (66%)
Female	20 (50%)	1 (33%)
Survival range (months)	2–48	5–12
Tumour location		
Temporal lobe	8 (21%)	2 (66%)
Frontal lobe	15 (39%)	
Parietal lobe	4 (11%)	
Occipital	4 (11%)	
More than one lobe	5 (13%)	1 (33%)
Multifocal	1 (3%)	
No data	1 (3%)	
Tumour recurrence		
Initial	38	3
Recurrent	7	1

**Table 2:** Summary of the number and proportion of *IDH*-wildtype and *IDH*-mutant glioblastoma patients with SNVs in genes in the RTK/Ras/PI(3)K, p53 DNA damage repair, WNT signalling, SHH, NOTCH, Rb, and G-protein pathways.

Pathway	*IDH*-wildtype				*IDH*-mutant			
	Initial		Recurrent		Initial		Recurrent	
	%	*N*	%	*N*	%	*N*	%	*N*
RTK/Ras/PI(3)K	79	30/38	43	3/7	66	2/3	0	0/1
p53 DNA damage repair	61	23/38	29	2/7	100	3/3	100	1/1
WNT signalling	58	22/38	57	4/7	33	1/3	100	1/1
SHH	16	6/38	0	0/7	0	0/3	0	0/1
NOTCH	8	3/38	0	0/7	0	0/3	0	0/1
Rb	5	2/38	0	0/7	0	0/3	0	0/1
G-protein	5	2/38	14	1/7	0	0/3	100	1/1

**Table 3:** Comparison of genes with SNVs identified in *IDH*-wildtype and *IDH*-mutant initial and recurrent tumours in the GBM cohort with those outlined by Barthel et al. [8], described for the five phases of gliomagenesis.

Gliomagenesis phases	Pathway	Common tumour genetic alterations (Barthel et al.)	*IDH* wildtype				*IDH*-mutant				
			Barthel et al.	GB-initial	GB-recurrent	GB-potentially pathogenic VUS	Barthel et al.	GB-initial	GB-recurrent	GB-potentially pathogenic VUS	Diagnostic panel (Y/N)
I: initial growth	IDH		—	*IDH1*	—	Y	*IDH1*	*IDH1*	—	Y	Y
	IDH		—	*IDH2*	—		*IDH2*	—	—		Y
	Rb		*CDK6*	*CDK6*	—		—	—	—		Y
	RTK/Ras/PI(3)K		*EGFR*	*EGFR*	*EGFR*	Y	—	—	—		Y
	RTK/Ras/PI(3)K		*MET*	*MET*	—		—	—	—		Y
	RTK/Ras/PI(3)K		*PDGFRA*	*PDGFRA*	—		*PDGFRA*	—	—		Y
	RTK/Ras/PI(3)K		*PIK3CA*	*PIK3CA*	—	Y	—	—	—		Y
	RTK/Ras/PI(3)K		*PIK3R1*	*PIK3R1*	—	Y	—	—	—		Y
	RTK/Ras/PI(3)K		*PTEN*	*PTEN*	*PTEN*	Y	—	—	—		Y
	WNT		*TERT*	*TERT*	*TERT*		—	—	—		Y
			*NF1*	—	—		—	—	—		Y
							*CTCF*				N
			*TET1*								N

II: oncogene-induced senescence	p53	*TP53*	*TP53*	*TP53*	—	Y	*TP53*	*TP53*	—	Y	Y
	p53	*CDKN2A*	*CDKN2A*	*CDKN2A*	—		—	—	—		Y
	p53		*PPM1D*	*PPM1D*	—	Y	—	—	—		Y
	Rb	*RB1*		*RB1*	—		—	—	—		Y
	RTK/Ras/PI(3)K	*BRAF*		*BRAF*	—	Y	—	—	—		Y
		*CDKN2B*	*CDKN2B*	—	—		—	—	—		Y
			*ACVR1*	—	—		—	—	—		Y

III: stressed growth	p53	*ATM*		*ATM*	*ATM*	Y	—	—			Y
	p53	*ATR*		*ATR*	—	Y	—	—	—		Y
		*MYC*		—	—		—	—	—		Y
		*CDK4*		—	—		—	—	—		Y
		*MDM2*		—	—		—	—	—		Y

IV: replicative senescence/crisis		*CHD5*									N
		*TREX1*									N
							*Terra*				*N*
		*RB1*	—	*RB1*	—		—	—	—		Y
	WNT	*TERT*	*TERT*	*TERT*	*TERT*		—	—	—		Y
	p53	*TP53*	*TP53*	*TP53*	—	Y	*TP53*	*TP53*	—	Y	Y
			*ATRX*	-	—		*ATRX*	—	—		Y
			—	*DAXX*	—	Y	*DAXX*	—	—		Y

V: immortalisation and dedifferentiation		*OLIG2*									N
		*SOX2*									N

GB-SNVs	G-proteins		—	*GNAS*	*GNAS*		—	—	*GNAS*		Y
	NOTCH		—	*NOTCH1*	—		—	—	—		Y
	NOTCH		—	*NOTCH2*	—		—	—	—		Y
	p53		—	*BRCA1*	*BRCA1*	Y	—	—	—		Y
	p53		—	*BRCA2*	*BRCA2*		—	*BRCA2*	—		Y
	p53		—	*BRPF3*	—		—	—	—		Y
	p53		—	*MDM4*	—		—	—	—		Y
	p53		—	*MSH2*	—		—	*MSH2*	—		Y
	p53		—	*MSH6*	—	Y	—	*MSH6*	—	Y	Y
	p53		—	*RAD50*	—		—	—	—		Y
	RTK/Ras/PI(3)K		—	*ALK*	—		—	—	—		Y
	RTK/Ras/PI(3)K		—	*CDH1*	—		—	*CDH1*	—		Y
	RTK/Ras/PI(3)K		—	*CSF1R*	*CSF1R*		—	*CSF1R*	—		Y
	RTK/Ras/PI(3)K		—	*FGFR2*	—	Y	—	—	—		Y
	RTK/Ras/PI(3)K		—	*FGFR3*	—		—	—	—		Y
	RTK/Ras/PI(3)K		—	*FGFR4*	—		—	—	—		Y
	RTK/Ras/PI(3)K		—	*FOXO3*	—		—	—	—		Y
	RTK/Ras/PI(3)K		—	*JAK2*	—	Y	—	—	—		Y
	RTK/Ras/PI(3)K		—	*KDR*	*KDR*		—	—	—		Y
	RTK/Ras/PI(3)K		—	*KLK1*	—		—	*KLK1*	—	Y	Y
	RTK/Ras/PI(3)K		—	*LZTR1*	—		—	—	—		Y
	RTK/Ras/PI(3)K		—	*MYB*	—	Y	—	—	—		Y
	RTK/Ras/PI(3)K		—	*NTRK2*	—		—	—	—		Y
	RTK/Ras/PI(3)K		—	*TSC2*	—	Y	—	*TSC2*	—	Y	Y
	SHH		—	*PTCH1*	—	Y	—	—	—		Y
	SHH		—	*PTCH2*	—		—	—	—		Y
	SHH		—	*SMO*	—	Y	—	—	—		Y
	WNT		—	*APC*	—		—	*APC*	—		Y
	WNT		—	*CREBBP*	—	Y	—	*CREBBP*	—	Y	Y
	WNT		—	*DICER1*	—		—	—	—		Y
	WNT		—	*KLF4*	—		—	—	—		Y
	WNT		—	*KMT2D*	—		—	—	—		Y

Risk mutations related to heritable diseases (Barthel et al. [8])		*TERC*									N
		*OBFC1*									N
		*POT1*									N
		*RTEL1*									N
		*TERT*	*TERT*	*TERT*	*TERT*		—	—	—		Y
		*TP53*	*TP53*	*TP53*	—	Y	*TP53*	*TP53*	—	Y	Y
		*NF1*	—	—	—		—	—	—		Y
		*NF2*	—	—	—		—	—	—		Y
		*CHK2 (CHEK2)*	—	*CHEK2*	—	Y	—	—	—		Y

Also included is a list of risk mutations related to heritable diseases. Genes identified with VUS that were possibly pathogenic in the GBM cohort are highlighted in bold.

**Table 4:** Summary of the proportion of initial and recurrent of *IDH*-wildtype and *IDH*-mutant glioblastoma patient tumours that had SNVs that could be assigned as potentially clinically actionable.

Gene	*IDH*-wildtype				*IDH*-mutant				Frequency in GBM (Sahm et al.)	Targeted agent (clinical trial)
	Initial tumour		Recurrent tumour		Initial tumour		Recurrent tumour			
	*N*	%	*N*	%	*N*	%	*N*	%	%	
*PIK3CA*	2/38	5	0/7	0	0/3	0	0/1	0	6.3	mTOR inhibitor; everolimus (NCT02449538); BKM120/everolimus (NCT01470209)
*PIK3R1*	2/38	5	0/7	0	0/3	0	0/1	0		mTOR inhibitor
*EGFR*	6/38	16	1/7	14	0/3	0	0/1	0	34	ABBV-221 (NCT02365662); naratinib (NCT01953926); AZD9291 (NCT02465060); EGFR-targeting antibodies, vaccines, TK inhibitors, osimertinib, poziotinib
*PDGFRA*	2/38	5	0/7	0	0/3	0	0/1	0	11	Dasatinib; nilotinib/Pazopanib (NCT02029001); MGCD516 (NCT02219711)
*BRAF*	1/38	3	0/7	0	0/3	0	0/1	0		Vemurafenib; MEK inhibitor
*PTEN*	11/38	29	1/7	14	0/3	0	0/1	0	32	INC280/BKM120 (NCT01870726); everolimus (NCT02449538); erlotinib, everolimus or dasatinib (NCT02233049); GSK2636771 (NCT01458067); BMN673 (NCT02286687); BKM120/everolimus (NCT01470209)
*BRCA1*	7/38	18	1/7	14	0/3	0	0/1	0		Olaparib
*BRCA2*	7/38	18	1/7	14	1/3	33	0/1	0		Olaparib
*PTCH1*	1/38	3	0/7	0	0/3	0	0/1	0		SMO inhibitor, sonidegib and vismodegib
*SMO*	3/38	8	0/7	0	0/3	0	0/1	0		SMO inhibitor, sonidegib and vismodegib
*ATR*	3/38	8	0/7	0	0/3	0	0/1	0		ATR inhibitor (BAY1895344)
*MSH6*	4/38	11	0/7	0	1/3	33	0/1	0	4.3	MK-3475 (NCT01876511)
*TP53*	7/38	18	0/7	0	3/3	100	0/1	0		
*CDKN2A*	3/38	8	0/7	0	0/3	0	0/1	0		
*RB1*	1/38	3	0/7	0	0/3	0	0/1	0		
*ATM*	6/38	16	1/7	14	0/3	0	0/1	0		

For particular genetic alterations, the proportion of glioblastomas (*n* = 47) with alterations in those genes, as recorded by Sahm et al. [30], is also provided. Also summarised are available and new therapeutic agents currently on trial in clinical studies targeting molecular aberrations.

## Data Availability

Data are available upon request from the Dept. of Neuropathology, Ruprecht-Karls University of Heidelberg.

## Supplementary Materials

Supplementary Materials Figure S1: oncoprint plot of mutations and copy number alterations identified in the TCGA-GBM dataset for 8 corresponding genes impacted by CNVs in the GBM cohort. Genes are represented as rows, and individual patients are represented as columns. The right barplot displays the number and type of alterations to each gene, categorised as AMP: high level amplification, GAIN: low level gain, HETLOSS: shallow deletion, HOMDEL: deep deletion, and MUT: SNV mutation event (green). Figure S2: oncoprint plot of mutations and copy number alterations identified in the TCGA-GBM dataset for the 21 corresponding genes impacted by VUS that were possibly pathogenic in the GBM cohort. Genes are represented as rows, and individual patients are represented as columns. The right barplot displays the number and type of alterations to each gene, categorised as AMP: high level amplification, GAIN: low level gain, HETLOSS: shallow deletion, HOMDEL: deep deletion, and MUT: SNV mutation event (green). Figure S3: oncoprint plot of mutations and copy number alterations identified in the TCGA-GBM dataset for 12 WNT/Notch/SHH pathway genes impacted by SNVs in the GBM cohort. Genes are represented as rows, and individual patients are represented as columns. The right barplot displays the number and type of alterations to each gene, categorised as AMP: high level amplification, GAIN: low level gain, HETLOSS: shallow deletion, HOMDEL: deep deletion, and MUT: SNV mutation event (green). Table S1: demographic data for the IDH-wildtype (*n* = 38) and IDH-mutant glioblastomas. Clinical records are for case ID, age, sex, tumour location on the MRI scan, IDH1 R132H hotspot mutation status, patient survival in months, and samples with matched initial and recurrent tumours. Table S2: list of the clinically relevant neuro-oncology genes that were analysed by the HTS-based diagnostic panel used in this study that was developed in Ruprecht Karl-University Heidelberg, Germany (see Sahm et al. [30]). Table S3: summary of the possibly pathogenic VUS identified in initial and recurrent IDH-wildtype and IDH-mutant glioblastoma tumours. The exonic nonsynonymous SNVs were predicted to be damaging by both LJB SIFT and FATHMM-MKL tools and had not been recorded by the 1000G database. Descriptive information for tumour, IDH status, genomic position, affected gene and pathway, available dbSNP and COSMIC identifiers, functional impacts predicted by LJB SIFT and FATHMM-MKL, and a shortened description from InterPro domain are provided. NA; not applicable (see Supplementary Tables Excel File). Table S4: summary of SNVs identified in initial tumours. Descriptive information for tumour, IDH status, genomic position, reference, and alternative variant alleles, affected gene, and pathway, ClinVar significance, functional impacts as predicted by LJB SIFT and FATHMM-MKL and available dbSNP and COSMIC identifiers and InterPro domain description are provided (see Supplementary Tables Excel File). Table S5: summary of SNVs identified in recurrent tumours. Descriptive information for tumour, IDH status, genomic position, reference and alternative variant alleles, affected gene and pathway, ClinVar significance, functional impacts as predicted by LJB SIFT and FATHMM-MKL and available dbSNP and COSMIC identifiers and InterPro domain description are provided (see Supplementary Tables Excel File). Table S6: summary of CNVs identified in initial and recurrent IDH-wildtype glioblastomas. CNV estimation is based on the read depth (%) of the variant (V) compared to a reference control (R; see Methods). Table S7: summary of the SNVs in TCGA-GBM dataset identified for the corresponding genes with VUS that were possibly pathogenic in the GB cohort. Descriptive information for tumour sample, gene, mutation type, amino acid change, genomic position, reference, and alternative variant alleles is provided (see Supplementary Tables Excel File). Table S8: number of cases in TCGA-GBM and GDC mutation datasets affected by mutations in the genes identified to have VUS that are possibly pathogenic in the GB cohort. According to TCGA, a total of 393 cases were tested for somatic mutations. TCGA-GBM comprises a small number of verified (*n* = 6) and ambiguous IDH-mutant cases (*n* = 2; see ). Table S9: number of cases in TCGA-GBM and GDC datasets affected by mutations in the WNT, notch, and SHH genes identified to have somatic mutations in the GB cohort. Table S10: mean and median survival time results of the survival analyses to test the impact of SNV burden on overall survival in MGMT methylated and unmethylated IDH-wildtype GBMs.
