# Supplementary material for: Clinically Actionable Insights into Initial and Matched Recurrent Glioblastomas to Inform Novel Treatment Approaches
Source: J Oncol. 2019 Dec 31;2019:4878547. doi: 10.1155/2019/4878547 (PMC7012245; doi:10.1155/2019/4878547)
Supplement: Supplementary Materials — Figure S1: oncoprint plot of mutations and copy number alterations identified in the TCGA-GBM dataset for 8 corresponding genes impacted by CNVs in the GBM cohort. Genes are represented as rows, and individual patients are represented as columns. The right barplot displays the number and type of alterations to each gene, categorised as AMP: high level amplification, GAIN: low level gain, HETLOSS: shallow deletion, HOMDEL: deep deletion, and MUT: SNV mutation event (green). Figure S2: oncoprint plot of mutations and copy number alterations identified in the TCGA-GBM dataset for the 21 corresponding genes impacted by VUS that were possibly pathogenic in the GBM cohort. Genes are represented as rows, and individual patients are represented as columns. The right barplot displays the number and type of alterations to each gene, categorised as AMP: high level amplification, GAIN: low level gain, HETLOSS: shallow deletion, HOMDEL: deep deletion, and MUT: SNV mutation event (green). Figure S3: oncoprint plot of mutations and copy number alterations identified in the TCGA-GBM dataset for 12 WNT/Notch/SHH pathway genes impacted by SNVs in the GBM cohort. Genes are represented as rows, and individual patients are represented as columns. The right barplot displays the number and type of alterations to each gene, categorised as AMP: high level amplification, GAIN: low level gain, HETLOSS: shallow deletion, HOMDEL: deep deletion, and MUT: SNV mutation event (green). Table S1: demographic data for the IDH-wildtype (n = 38) and IDH-mutant glioblastomas. Clinical records are for case ID, age, sex, tumour location on the MRI scan, IDH1 R132H hotspot mutation status, patient survival in months, and samples with matched initial and recurrent tumours. Table S2: list of the clinically relevant neuro-oncology genes that were analysed by the HTS-based diagnostic panel used in this study that was developed in Ruprecht Karl-University Heidelberg, Germany (see Sahm et al. [30]). Table S3: sum [file 4878547.f1.zip › 4878547.f1/Ellis et al 281019 Supplementary Materials.pdf]

SUPPLEMENTARY MATERIALS

FIGURES

Figure S1. Oncoprint plot of mutations and copy number alterations identified in the TCGA-GBM dataset for 8 corresponding genes impacted by CNVs in the GBM cohort. Genes are represented as rows and individual patients are represented as columns. The right barplot displays the number and type of alterations to each gene, categorised as: AMP: High level amplification; GAIN: low level gain; HETLOSS: shallow deletion; HOMDEL: deep deletion and MUT: SNV mutation event (green).

Figure S2. Oncoprint plot of mutations and copy number alterations identified in the TCGA-GBM dataset for the 21 corresponding genes impacted by VUS that were possibly pathogenic in the GBM cohort. Genes are represented as rows and individual patients are represented as columns. The right barplot displays the number and type of alterations to each gene, categorised as: AMP: High level amplification; GAIN: low level gain; HETLOSS: shallow deletion; HOMDEL: deep deletion and MUT: SNV mutation event (green).

Figure S3. Oncoprint plot of mutations and copy number alterations identified in the TCGA-GBM dataset for 12 WNT/Notch/SHH pathway genes impacted by SNVs in the GBM cohort. Genes are represented as rows and individual patients are represented as columns. The right barplot displays the number and type of alterations to each gene, categorised as: AMP: High level amplification; GAIN: low level gain; HETLOSS: shallow deletion; HOMDEL: deep deletion and MUT: SNV mutation event (green).

TABLES

Table S1. Demographic data for the *IDH*-wildtype (n=38) and *IDH*-mutant glioblastomas. Clinical records are for case ID, age, sex, tumour location on the MRI scan, *IDH1* R132H hotspot mutation status, patient survival in months and samples with matched initial and recurrent tumours.

Patient Case ID	Age	Sex	Tumour Location	*IDH*	Survival (months)	Matched Initial & Recurrent samples
8	19	F	right fron/temp	mut	12	Y
35	58	M	right temporal	mut	5	
39	50	M	right temporal	mut	6	
1	59	M	left frontal	WT	11	Y
2	47	F	right temporal	WT	17	Y
3	48	F	right fron/temp	WT	2	Y
4	60	F	left frontal	WT	13	Y
5	69	F	right frontal	WT	31	Y
6	65	F	right frontal	WT	24	
7	50	M	left temporal	WT	14	Y
9	72	F	right pariet/temp	WT	13	
10	61	F	right temporal	WT	2	
11	16	M	left parietal	WT	-	
16	52	M	right parietal	WT	48	
17	41	F	left frontal	WT	20	
18	49	M	left frontal	WT	5	
19	66	F	left temporal	WT	21	
20	43	M	left frontal	WT	-	
21	65	M	left parietal	WT	13	
22	46	F	left temporal	WT	11	
23	50	M	left temp/parietal	WT	38	
24	78	M	N.d.	WT	-	
25	60	M	right occipital	WT	11	
26	59	F	left temporal	WT	12	
27	67	F	multifocal	WT	13	
28	59	F	left parietal	WT	11	
29	63	M	left occipital	WT	-	
30	41	M	left occipital	WT	15	
31	52	F	left frontal	WT	23	
32	51	M	right temporal	WT	-	
33	75	M	left parieto-occ	WT	4	
34	63	M	left temporal	WT	8	
36	43	F	right frontal	WT	10	Y
37	49	M	left frontal	WT	40	
38	50	F	left parieto-occ	WT	14	
40	35	F	right frontal	WT	alive (50+)	
41	38	F	right frontal	WT	23	
43	68	M	right frontal	WT	10	
44	40	M	right frontal	WT	5	
45	74	F	right frontal	WT	10	
46	44	M	right occipital	WT	11	

N.d. : no data; "-" : Date of death was not listed on the clinical follow-up. Mut = mutant; WT = wildtype

Table S2. List of the clinically relevant neuro-oncology genes that were analysed by the HTS-based diagnostic panel used in this study that was developed in Ruprecht-Karls-University Heidelberg, Germany (see Sahm et al. 2016).

**Heidelberg diagnostic panel genes**					
ABL1	*CDKN2A*	*GNAS*	*MGMT*	*PPM1D*	*TRAF7*
ACVR1	*CDKN2B*	*H2AFX*	*MLH1*	*PRKAR1A*	*TSC1*
AKT1	*CDKN2C*	*H3F3A*	*MLL2*	*PTCH1*	*TSC2*
AKT2	*CHEK2*	*HDAC2*	*MPL*	*PTCH2*	*VHL*
AKT3	*CIC*	*HIST1H3B*	*MRE11A*	*PTEN*	
ALK	*CREBBP*	*HIST1H3C*	*MSH2*	*PTPN11*	
APC	*CSF1R*	*HNF1A*	*MSH6*	*RAD50*	
ARID1A	*CTNNB1*	*HRAS*	*MYB*	*RAF1*	
ARID1B	*D2HGDH*	*IDH1*	*MYBL1*	*RB1*	
ARID2	*DAXX*	*IDH2*	*MYC*	*RET*	
ATM	*DDX3X*	*IDO2*	*MYCN*	*SETD2*	
ATR	*DICER1*	*JAK2*	*MYL1*	*SMAD4*	
ATRX	*EGFR*	*JAK3*	*NBN*	*SMARCA2*	
BCOR	*EZH2*	*KDM6A*	*NDRG2*	*SMARCA4*	
BRAF	*FBXW7*	*KDR*	*NF1*	*SMARCB1*	
BRCA1	*FGFR1*	*KIAA0182*	*NF2*	*SMARCD1*	
BRCA2	*FGFR2*	*KIT*	*NOTCH1*	*SMARCD2*	
BRPF1	*FGFR3*	*KLF4*	*NOTCH2*	*SMARCE1*	
BRPF3	*FGFR4*	*KLK1*	*NRAS*	*SMO*	
C11ORF95	*FLT3*	*KRAS*	*NTRK2*	*STAG2*	
CCND1	*FOXO3*	*LDB1*	*PCDH8*	*SUFU*	
CCND2	*FUBP1*	*LZTR1*	*PDGFRA*	*TBR1*	
CDH1	*GABRA6*	*MDM2*	*PIK3C2G*	*TCF4*	
CDK4	*GNA11*	*MDM4*	*PIK3CA*	*TERT*	
CDK6	*GNAQ*	*MET*	*PIK3R1*	*TP53*	

Table S3. Summary of the possibly pathogenic VUS identified in initial and recurrent *IDH*-wildtype and -*IDH*-mutant glioblastoma tumours. The exonic non-synonymous SNVs were predicted to be damaging by both LJB SIFT and FATHMM-MKL tools and had not been recorded by the 1000G database. Descriptive information for tumour, *IDH* status, genomic position, affected gene and pathway, available dbSNP and COSMIC identifiers, functional impacts predicted by LJB SIFT and FATHMM-MKL and a shortened description from InterPro domain are provided. NA; not applicable. (See Supplementary Tables Excel File)

Table S4. Summary of SNVs identified in initial tumours. Descriptive information for tumour, *IDH* status, genomic position, reference and alternative variant alleles, affected gene and pathway, ClinVar significance, functional impacts as predicted by LJB SIFT and FATHMM-MKL and available dbSNP and COSMIC identifiers and InterPro domain description are provided. (See Supplementary Tables Excel File)

Table S5. Summary of SNVs identified in recurrent tumours. Descriptive information for tumour, *IDH* status, genomic position, reference and alternative variant alleles, affected gene and pathway, ClinVar significance, functional impacts as predicted by LJB SIFT and FATHMM-MKL and available dbSNP and COSMIC identifiers and InterPro domain description are provided. (See Supplementary Tables Excel File)

Table S6. Summary of CNVs identified in initial and recurrent *IDH*-wildtype glioblastomas. CNV estimation is based on the read depth (%) of the variant (V) compared to a reference control (R; see Methods).

**Sample**	**R / V**	***KDR***	***TERT***	***SMARCA4***	***EGFR***	***KMT2D***	***GNAS***	***SETD2***	***BRCA2***
**1a**	**R**	238 (43%)	215 (48%)	207 (30%)					
	**V**	311 (57%)	229 (52%)	**479 (70%)**					
**1b**	**R**	106 (48%)	255 (69%)	119 (67%)					
	**V**	117 (52%)	**116 (31%)**	**59 (33%)**					
**2a**	**R**				391 (5%)				
	**V**				**6917 (95%)**				
**2b**	**R**				556 (8%)				
	**V**				**6676 (92%)**				
**3a**	**R**					159 (51%)	98 (51%)		
	**V**					152 (49%)	95 (49%)		
**3b**	**R**					90 (41%)	46 (32%)		
	**V**					132 (59%)	**99 (68%)**		
**4a**	**R**							465 (54%)	
	**V**							392 (46%)	
**4b**	**R**							206 (62%)	
	**V**							**128 (38%)**	
**7a**	**R**		229 (55%)						
	**V**		190 (45%)						
**7b**	**R**		346 (70%)						
	**V**		**145 (30%)**						
**36a**	**R**					614 (43%)			372 (68%)
	**V**					824 (57%)			**172 (32%)**
**36b**	**R**					826 (50%)			76 (17%)
	**V**					824 (50%)			**360 (83%)**

Table S7. Summary of the SNVs in TCGA-GBM dataset identified for the corresponding genes with VUS that were possibly pathogenic in the GB cohort. Descriptive information for tumour sample, gene, mutation type, amino acid change, genomic position, reference and alternative variant alleles is provided. (See Supplementary Tables Excel File)

Table S8. Number of cases in TCGA-GBM and GDC mutation datasets affected by mutations in the genes identified to have VUS that are possibly pathogenic in the GB cohort. According to TCGA a total of 393 cases were tested for somatic mutations. TCGA-GBM comprises a small number of verified (n=6) and ambiguous *IDH*-mutant cases (n=2; see https://tcga-data.nci.nih.gov/docs/publications/gbm_exp/IDH1-Mutated_Samples.txt).

**Gene**	**Pathway**	**Number of Affected Cases in TCGA-GBM cohort (%)**	**Number of Affected Cases across the GDC**	**Number of Mutations in TCGA-GBM cohort**
PTEN	RTK/Ras/PI(3)K	137 / 393 (34.86%)	1,005 / 10,202 (9.85%)	112
TP53	P53	124 / 393 (31.55%)	4,008 / 10,202 (39.29%)	100
EGFR	RTK/Ras/PI(3)K	106 / 393 (26.97%)	548 / 10,202 (5.37%)	74
PIK3R1	RTK/Ras/PI(3)K	43 / 393 (10.94%)	540 / 10,202 (5.29%)	40
PIK3CA	RTK/Ras/PI(3)K	40 / 393 (10.18%)	1,403 / 10,202 (13.75%)	38
IDH1	*IDH*	26 / 393 (6.62%)	566 / 10,202 (5.55%)	5
PTCH1	SHH	14 / 393 (3.56%)	368 / 10,202 (3.61%)	18
CREBBP	WNT	14 / 393 (3.56%)	578 / 10,202 (5.67%)	19
MSH6	P53	12 / 393 (3.05%)	254 / 10,202 (2.49%)	16
BRCA1	P53	11 / 393 (2.80%)	332 / 10,202 (3.25%)	16
BRAF	RTK/Ras/PI(3)K	10 / 393 (2.54%)	813 / 10,202 (7.97%)	6
DAXX	RTK/Ras/PI(3)K	9 / 393 (2.29%)	201 / 10,202 (1.97%)	10
ATM	P53	8 / 393 (2.04%)	618 / 10,202 (6.06%)	13
TSC2	RTK/Ras/PI(3)K	8 / 393 (2.04%)	326 / 10,202 (3.20%)	8
PPM1D	P53	7 / 393 (1.78%)	139 / 10,202 (1.36%)	9
FGFR2	RTK/Ras/PI(3)K	6 / 393 (1.53%)	314 / 10,202 (3.08%)	8
JAK2	RTK/Ras/PI(3)K	5 / 393 (1.27%)	221 / 10,202 (2.17%)	7
MYB	RTK/Ras/PI(3)K	5 / 393 (1.27%)	197 / 10,202 (1.93%)	5
SMO	SHH	4 / 393 (1.02%)	161 / 10,202 (1.58%)	5
KLK1	RTK/Ras/PI(3)K	2 / 393 (0.51%)	105 / 10,202 (1.03%)	2
CHEK2	P53	1 / 393 (0.25%)	158 / 10,202 (1.55%)	2

Table S9. Number of cases in TCGA-GBM and GDC datasets affected by mutations in the WNT, Notch and SHH genes identified to have somatic mutations in the GB cohort.

Gene	Pathway	Number of Affected Cases TCGA-GBM	Number of Affected Cases Across the GDC	Number of Mutations
APC	WNT	18 / 393 (4.58%)	893 / 10,202	27
CREBBP	WNT	14 / 393 (3.56%)	578 / 10,202	19
KMT2D	WNT	12 / 393 (3.05%)	1,140 / 10,202	17
TERT	WNT	11 / 393 (2.80%)	194 / 10,202	17
DICER1	WNT	9 / 393 (2.29%)	345 / 10,202	15
TCF4	WNT	3 / 393 (0.76%)	305 / 10,202	3
KLF4	WNT	1 / 393 (0.25%)	109 / 10,202	1
PTCH1	SHH	14 / 393 (3.56%)	368 / 10,202	18
PTCH2	SHH	7 / 393 (1.78%)	223 / 10,202	8
SMO	SHH	4 / 393 (1.02%)	161 / 10,202	5
NOTCH1	NOTCH	1 / 393 (0.25%)	513 / 10,202	1
NOTCH2	NOTCH	16 / 393 (4.07%)	400 / 10,202	20

Table S10. Mean and median survival time results of the survival analyses to test the impact of SNV burden on overall survival in *MGMT* methylated and unmethylated *IDH*-wildtype GBMs.

MGMT status	Total Variation in SNVs		Mean[a]				Median			
		N	Estimate	Std. Error	95% Confidence Interval		Estimate	Std. Error	95% Confidence Interval	
					Lower Bound	Upper Bound			Lower Bound	Upper Bound
**Methylated**	<= 4	10	26.4	4.684	17.218	35.582	23	3.162	16.802	29.198
	>= 5	5	9.2	2.634	4.037	14.363	10	5.477	0	20.735
	Overall	15	20.667	3.841	13.138	28.195	17	5.152	6.901	27.099
**Unmethylated**	<= 4	10	16.5	2.802	11.009	21.991	13	1.186	10.676	15.324
	>= 5	8	9.75	1.567	6.679	12.821	11	2.143	6.799	15.201
	Overall	18	13.5	1.849	9.875	17.125	11	0.795	9.441	12.559

a. Estimation is limited to the largest survival time if it is censored
