# Supplementary figures and images for: Clinically Actionable Insights into Initial and Matched Recurrent Glioblastomas to Inform Novel Treatment Approaches
Source: J Oncol. 2019 Dec 31;2019:4878547. doi: 10.1155/2019/4878547 (PMC7012245; doi:10.1155/2019/4878547)

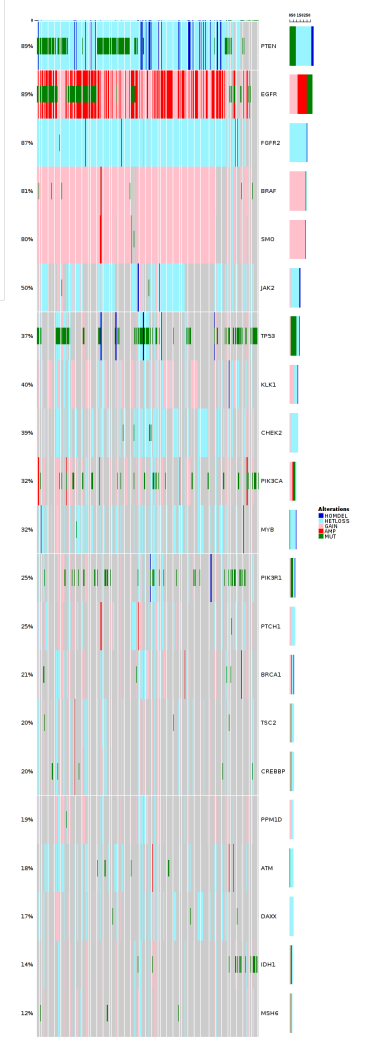

Supplement: Supplementary Materials — Figure S1: oncoprint plot of mutations and copy number alterations identified in the TCGA-GBM dataset for 8 corresponding genes impacted by CNVs in the GBM cohort. Genes are represented as rows, and individual patients are represented as columns. The right barplot displays the number and type of alterations to each gene, categorised as AMP: high level amplification, GAIN: low level gain, HETLOSS: shallow deletion, HOMDEL: deep deletion, and MUT: SNV mutation event (green). Figure S2: oncoprint plot of mutations and copy number alterations identified in the TCGA-GBM dataset for the 21 corresponding genes impacted by VUS that were possibly pathogenic in the GBM cohort. Genes are represented as rows, and individual patients are represented as columns. The right barplot displays the number and type of alterations to each gene, categorised as AMP: high level amplification, GAIN: low level gain, HETLOSS: shallow deletion, HOMDEL: deep deletion, and MUT: SNV mutation event (green). Figure S3: oncoprint plot of mutations and copy number alterations identified in the TCGA-GBM dataset for 12 WNT/Notch/SHH pathway genes impacted by SNVs in the GBM cohort. Genes are represented as rows, and individual patients are represented as columns. The right barplot displays the number and type of alterations to each gene, categorised as AMP: high level amplification, GAIN: low level gain, HETLOSS: shallow deletion, HOMDEL: deep deletion, and MUT: SNV mutation event (green). Table S1: demographic data for the IDH-wildtype (n = 38) and IDH-mutant glioblastomas. Clinical records are for case ID, age, sex, tumour location on the MRI scan, IDH1 R132H hotspot mutation status, patient survival in months, and samples with matched initial and recurrent tumours. Table S2: list of the clinically relevant neuro-oncology genes that were analysed by the HTS-based diagnostic panel used in this study that was developed in Ruprecht Karl-University Heidelberg, Germany (see Sahm et al. [30]). Table S3: sum [file 4878547.f1.zip › 4878547.f1/FigS1_TGCA_mutations.jpg]

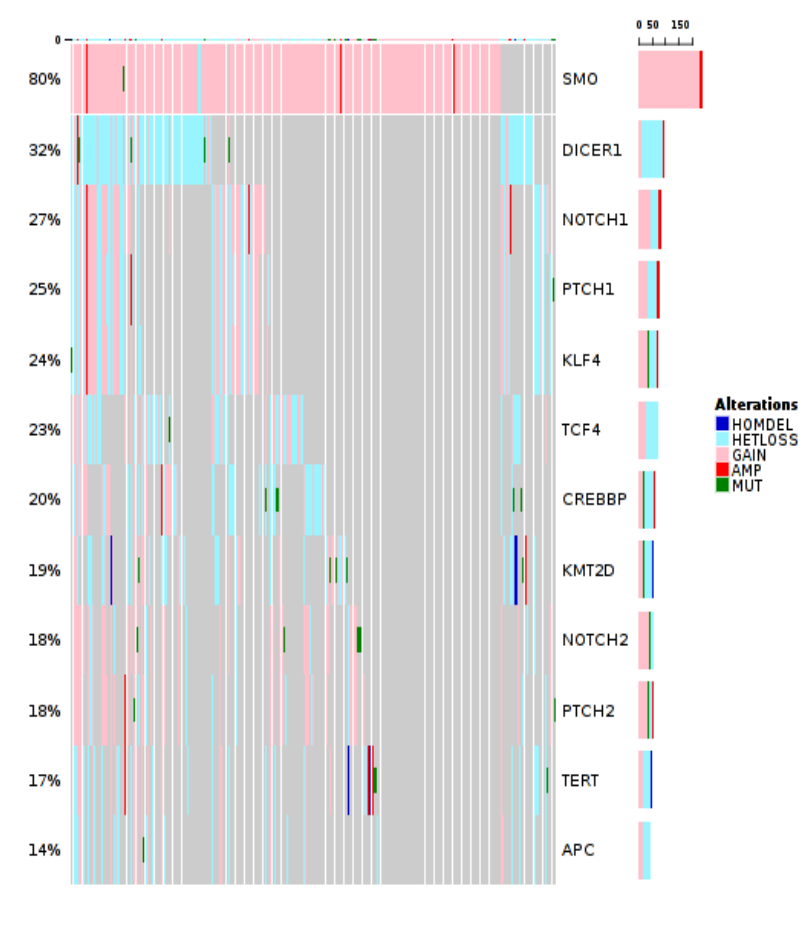

Supplement: Supplementary Materials — Figure S1: oncoprint plot of mutations and copy number alterations identified in the TCGA-GBM dataset for 8 corresponding genes impacted by CNVs in the GBM cohort. Genes are represented as rows, and individual patients are represented as columns. The right barplot displays the number and type of alterations to each gene, categorised as AMP: high level amplification, GAIN: low level gain, HETLOSS: shallow deletion, HOMDEL: deep deletion, and MUT: SNV mutation event (green). Figure S2: oncoprint plot of mutations and copy number alterations identified in the TCGA-GBM dataset for the 21 corresponding genes impacted by VUS that were possibly pathogenic in the GBM cohort. Genes are represented as rows, and individual patients are represented as columns. The right barplot displays the number and type of alterations to each gene, categorised as AMP: high level amplification, GAIN: low level gain, HETLOSS: shallow deletion, HOMDEL: deep deletion, and MUT: SNV mutation event (green). Figure S3: oncoprint plot of mutations and copy number alterations identified in the TCGA-GBM dataset for 12 WNT/Notch/SHH pathway genes impacted by SNVs in the GBM cohort. Genes are represented as rows, and individual patients are represented as columns. The right barplot displays the number and type of alterations to each gene, categorised as AMP: high level amplification, GAIN: low level gain, HETLOSS: shallow deletion, HOMDEL: deep deletion, and MUT: SNV mutation event (green). Table S1: demographic data for the IDH-wildtype (n = 38) and IDH-mutant glioblastomas. Clinical records are for case ID, age, sex, tumour location on the MRI scan, IDH1 R132H hotspot mutation status, patient survival in months, and samples with matched initial and recurrent tumours. Table S2: list of the clinically relevant neuro-oncology genes that were analysed by the HTS-based diagnostic panel used in this study that was developed in Ruprecht Karl-University Heidelberg, Germany (see Sahm et al. [30]). Table S3: sum [file 4878547.f1.zip › 4878547.f1/FigS2_WNT,NOTCH,SHH.jpg]

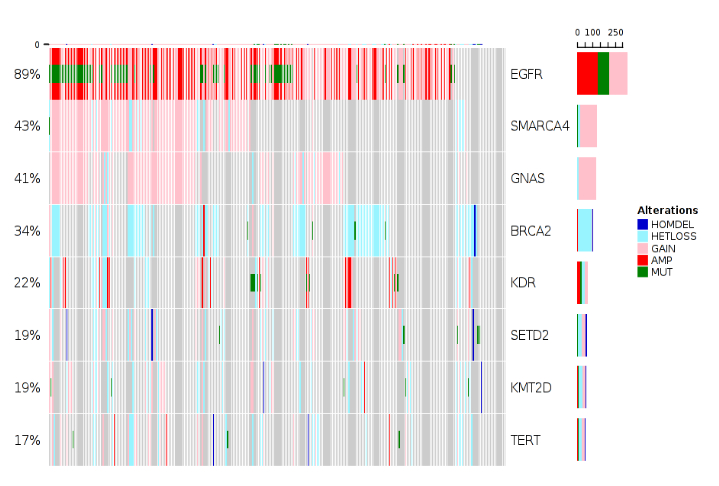

Supplement: Supplementary Materials — Figure S1: oncoprint plot of mutations and copy number alterations identified in the TCGA-GBM dataset for 8 corresponding genes impacted by CNVs in the GBM cohort. Genes are represented as rows, and individual patients are represented as columns. The right barplot displays the number and type of alterations to each gene, categorised as AMP: high level amplification, GAIN: low level gain, HETLOSS: shallow deletion, HOMDEL: deep deletion, and MUT: SNV mutation event (green). Figure S2: oncoprint plot of mutations and copy number alterations identified in the TCGA-GBM dataset for the 21 corresponding genes impacted by VUS that were possibly pathogenic in the GBM cohort. Genes are represented as rows, and individual patients are represented as columns. The right barplot displays the number and type of alterations to each gene, categorised as AMP: high level amplification, GAIN: low level gain, HETLOSS: shallow deletion, HOMDEL: deep deletion, and MUT: SNV mutation event (green). Figure S3: oncoprint plot of mutations and copy number alterations identified in the TCGA-GBM dataset for 12 WNT/Notch/SHH pathway genes impacted by SNVs in the GBM cohort. Genes are represented as rows, and individual patients are represented as columns. The right barplot displays the number and type of alterations to each gene, categorised as AMP: high level amplification, GAIN: low level gain, HETLOSS: shallow deletion, HOMDEL: deep deletion, and MUT: SNV mutation event (green). Table S1: demographic data for the IDH-wildtype (n = 38) and IDH-mutant glioblastomas. Clinical records are for case ID, age, sex, tumour location on the MRI scan, IDH1 R132H hotspot mutation status, patient survival in months, and samples with matched initial and recurrent tumours. Table S2: list of the clinically relevant neuro-oncology genes that were analysed by the HTS-based diagnostic panel used in this study that was developed in Ruprecht Karl-University Heidelberg, Germany (see Sahm et al. [30]). Table S3: sum [file 4878547.f1.zip › 4878547.f1/FigS3_CNV.jpg]
